# Evaluating the Implementation of an Intervention to Improve Postpartum Contraception in Tanzania: A Qualitative Study of Provider and Client Perspectives

**DOI:** 10.9745/GHSP-D-19-00365

**Published:** 2020-06-30

**Authors:** Kristy Hackett, Sarah Huber-Krum, Joel M. Francis, Leigh Senderowicz, Erin Pearson, Hellen Siril, Nzovu Ulenga, Iqbal Shah

**Affiliations:** a Department of Global Health and Population, Harvard T.H. Chan School of Public Health, Boston, MA, USA.; b Management and Development for Health, Dar es Salaam, Tanzania.; c Department of Family Medicine and Primary Care, School of Clinical Medicine, Faculty of Health Sciences, University of the Witwatersrand, Johannesburg, South Africa.; d Technical Innovation and Evidence, Ipas, Chapel Hill, NC, USA.

## Abstract

**Background:**

This qualitative study assessed implementation of the Postpartum Intrauterine Device (PPIUD) Initiative in Tanzania, a country with high rates of unintended pregnancy and low contraceptive prevalence. The PPIUD Initiative was implemented to reduce unmet need for contraception among new mothers through postpartum family planning counseling delivered during antenatal care and offering PPIUD insertion immediately following birth.

**Methods:**

We used the implementation outcomes framework and an ecological framework to analyze in-depth interviews with providers (N=15) and women (N=47) participating in the initiative. We applied a multistage coding protocol and used thematic content analysis to identify the factors influencing implementation.

**Results:**

Both women and providers were enthusiastic and receptive to the PPIUD Initiative. Health system and resource constraints made adoption and fidelity to the intended intervention challenging. Many providers questioned the sustainability of the initiative, and most agreed that changes to the initiative’s design (e.g., additional training opportunities, improved staffing, and availability of PPIUD supplies) would strengthen future iterations of the initiative. According to women, interpersonal aspects of care varied, with some women reporting rushed or incomplete counseling or an emphasis on the PPIUD over other methods. The perception that some providers treat older married women more favorably suggests that fidelity to the intended PPIUD Initiative was not uniformly achieved.

**Conclusions:**

Study findings inform initiatives seeking to develop and adopt postpartum family planning programs and enhance program implementation. A comprehensive needs assessment to evaluate feasibility and identify potential adaptations for the local context is recommended. Training and supervision to improve interpersonal aspects of care, including an emphasis on patient-centered counseling, informed choice, and respectful and nondiscriminatory service delivery should be integrated into future postpartum family planning initiatives.

## Abstract (precis)

Training and supervision to improve interpersonal aspects of care, including an emphasis on patient-centered counseling, informed choice, and respectful and nondiscriminatory service delivery, should be integrated into future postpartum family planning initiatives.

## Teaser

**Key Findings:**

- Constraints on staff, time, and supplies and challenges with referrals influenced implementation outcomes and threatened sustainability.
- Women reported that interpersonal aspects of care varied.
- Providers reported that additional training opportunities, improved staffing, and increased availability of PPIUD supplies would strengthen future initiatives.

**Key Implications:**

We recommend that PPFP program imple-menters consider:

- Assessing the feasibility of integrating PPFP counseling into existing antenatal care services before program implementation
- Ensuring that future initiatives emphasize patient-centered PPFP counseling, informed choice, and respectful and nondiscriminatory service delivery
- Implementing a stronger interfacility performance and quality improvement system to strengthen coordination
- Using different models of PPFP counseling to help alleviate provider workload

## INTRODUCTION

Unintended and mistimed pregnancies are pressing global public health concerns due to their associations with increased maternal, newborn, and child morbidity and mortality.^1^^–^^4^ The postpartum period represents a critical window of opportunity to ensure healthy timing and spacing of subsequent pregnancies and to address unmet need for family planning.^5^ Despite increased attention to the postpartum period as an opportunity for family planning interventions, recent estimates from 57 countries show that 62% of women have an unmet need for contraception immediately after delivery, and 40% have an unmet need within the first year after birth.^6^ Postpartum family planning (PPFP) initiatives that respect women’s right to make full and informed choices have the potential to help meet these needs.

Where available, PPFP counseling is part of postnatal care (PNC) services.^7^ However, PPFP counseling is not consistently provided, and PNC often disproportionally focuses on the well-being of the newborn rather than the mother. Additionally, in many low-income settings, a host of socioeconomic obstacles prevent mothers from returning to facilities for postnatal check-ups; therefore, opportunities for PPFP are often missed.^8^ One strategy to overcome this challenge is to provide PPFP counseling during antenatal care (ANC), which is more widely used than PNC, followed by immediate insertion of the intrauterine device (IUD) after delivery (within 10 minutes following delivery of the placenta or within 48 hours) to women who opt for this method. The copper IUD is widely accepted as an effective, long-acting (up to 12 years), reversible method of contraception and is particularly convenient when inserted immediately after birth.^9^ Other benefits of the postpartum IUD (PPIUD) are that it can be inserted after either vaginal or cesarean delivery, does not interfere with breastfeeding, and can be used by women who have HIV.^9^ Several systematic reviews have established the safety and effectiveness of PPIUD insertion.^10^^,^^11^

Immediate PPIUD insertion is a specialized technique that differs from interval IUD insertion, and thus requires additional hands-on didactic training and specialized equipment.^12^ Due to the early postpartum timing of the procedure and the rapid change in the uterus during this time, expulsion rates for immediate PPIUD insertions are higher than for interval insertions.^12^ According to a recent review, expulsion rates vary by timing of IUD placement, ranging from 1.9% with interval placements (greater than 4 weeks postpartum), 10.0% for immediate placements within 10 minutes following placental delivery, and 29.7% for placements between 10 minutes to 4 weeks postpartum.^13^ For these reasons, it is recommended that women who opt for PPIUD insertion are counseled regarding the increased expulsion risk, as well as signs and symptoms of expulsion.^12^

Recent improvements in institutional delivery rates across sub-Saharan Africa^14^ make prenatal contraceptive counseling and PPIUD a promising PPFP option among populations likely to receive ANC and/or deliver in facilities but unlikely to return for care in the postpartum period. In Tanzania, institutional delivery rates have increased substantially in recent decades (44% to 63% between 1999 and 2016) and uptake of ANC from a skilled provider is almost universal (98% in 2016), yet PNC coverage has been slow to improve.^15^ Only 37% of mothers receive a postnatal checkup after their most recent live birth^15^ and the quality of postnatal services is often low.^16^

Therefore, providing prenatal contraceptive counseling and offering PPIUD insertion is a desirable intervention in Tanzania, where the government recently committed to increasing the availability of modern contraception at all levels of the health system from 40% in 2012 to 70% by 2020.^17^ Tanzania’s modern contraceptive prevalence rate (mCPR) is 31%, and more than a quarter of reproductive-age women have an unmet need for modern contraception.^17^ Despite the known benefits of PPIUD, uptake of the IUD, regardless of insertion timing, is low in Tanzania (less than 1% among reproductive-age women).^15^ Programs providing PPIUD services are just beginning to emerge in low- and middle-income countries (LMICs); consequently, there is a gap in the literature on PPIUD programs, with few published evaluations and limited research on implementation processes.^18^ This study helps to fill this gap by assessing the factors influencing implementation of a novel PPIUD Initiative in Tanzania.

## PPIUD INITIATIVE DESCRIPTION AND ACHIEVEMENTS

**FIGURE 1.**
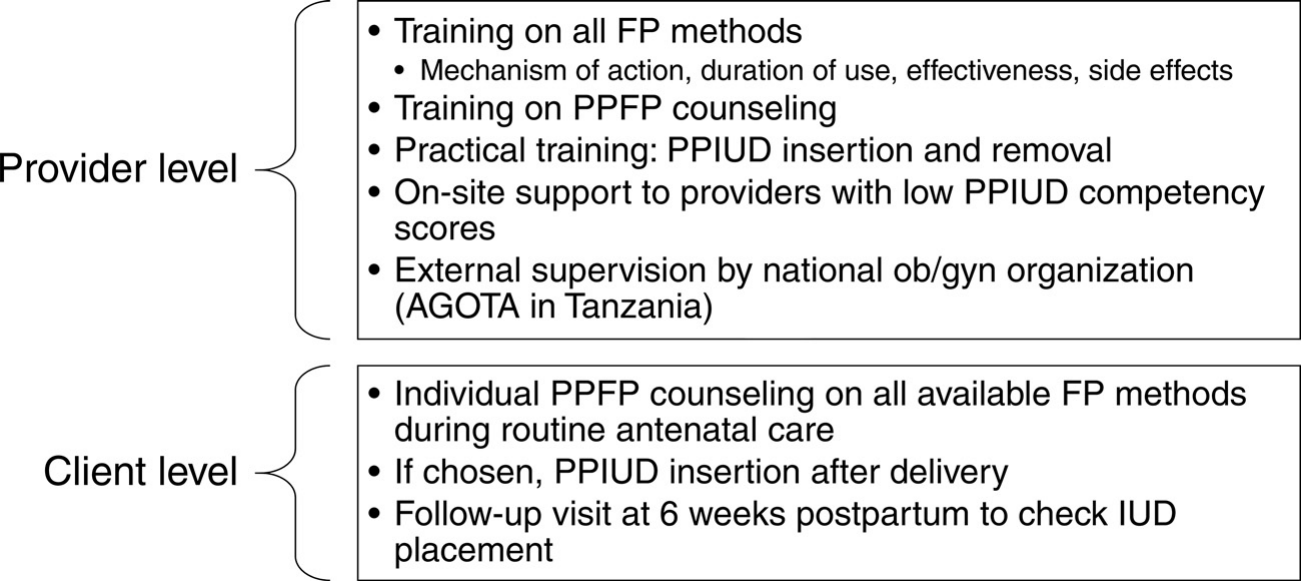
Provider- and Client-Level Interventions for the PPIUD Initiative Abbreviations: AGOTA, Association of Gynecologists and Obstetricians of Tanzania; FP, family planning; IUD, intrauterine device; ob/gyn, obstetrics/gynecology; PPFP, postpartum family planning; PPIUD, postpartum intrauterine device.

In 2013, the International Federation of Gynecology and Obstetrics (FIGO) launched an initiative to institutionalize PPIUD services in Sri Lanka, followed by Tanzania, Nepal, India, Kenya, and Bangladesh in 2015.^19^ Through this initiative, clinic and hospital staff in select facilities received training on the provision of PPFP counseling and PPIUD insertion techniques as novel services. To promote sustainability, the project was designed for implementation within existing maternity services, and current staff were intended to deliver PPFP counseling and PPIUD insertion rather than recruiting new health workers. Facilities were selected on the basis of having a large annual obstetric caseload (>5000 deliveries), a large number of providers and medical trainees, and no PPIUD services already provided.^19^ In each country, the project was implemented through national professional societies or colleges to encourage ownership by in-country obstetricians and gynecologists. Although the project design and budget allowed for country-specific tailoring, the same general model was implemented in each country. This model included a dedicated project management team, a coordinator at each participating facility, a Data Safety Monitoring Committee, and national steering groups who provided clinical and technical guidance.^19^ Further details on the PPIUD Initiative design and components are published elsewhere.^19^ We present key elements and inputs of the PPIUD Initiative in Table 1 and summarize intervention components at the provider and client levels in Figure 1.

**TABLE 1.** Key Elements and Inputs Intended for Implementation of the PPIUD Initiative in 6 Countries

**Elements and Inputs**	**Description**
Training: “Train-the-trainer” model: Training cascaded from master trainers to existing eligible providers at selected health facilities	Counseling: Prenatal counseling on available family planning methods with an emphasis on PPFP using standard training methods (e.g., GATHER model) Included information on the advantages of PPIUD Open discussion about providers’ views of PPIUD to address any prejudices Role play, case scenarios Providers encouraged to use counseling aids (e.g., leaflets, posters, flipcharts, and videos) PPIUD insertion and removal: Theoretical (classroom-based) training and practical sessions using Mama-U postpartum uterus models Refresher trainings offered as needed Regular training of new staff rotating in
Equipment and supplies	Mama-U models Copper T IUDs Long-handled 33 cm curved Kelly forceps
National coordination	Implementation was coordinated through national professional societies or colleges to encourage local ownership National societies set up steering groups for clinical and technical guidance
Structures established to facilitate implementation	Dedicated project management team Facility-level project coordinators Data Safety Monitoring Committees
PPIUD counseling and insertion services delivered by trained providers	Integrated within existing maternity services Prenatal counseling on all available contraception methods with an emphasis on PPFP, and the advantages of PPIUD as a safe, effective, and reversible long-acting method Consent forms provided during prenatal visits Stickers placed on women’s case files to identify consenting women at delivery Women who did not receive prenatal counseling could be counseled during early labor or the postnatal period to ensure insertion within 48 hours if PPIUD was desired
Monitoring and evaluation	Data collection officers collected information on counseling, consent, PPIUD, and follow-up for women delivering in participating facilities

Abbreviations: GATHER, greet, ask, tell, help, explain, and return; IUD, intrauterine device; PPFP, postpartum family planning; PPIUD, postpartum intrauterine device.

### Tanzania PPIUD Initiative

In Tanzania, the PPIUD Initiative was first implemented in 6 hospitals. However, 1 facility was dropped because it had an ongoing PPIUD intervention. Therefore, the study was conducted in 5 hospitals. FIGO and their local affiliate, the Association of Gynecologists and Obstetricians of Tanzania (AGOTA): (1) trained maternity care providers at tertiary/teaching hospitals on PPFP counseling, PPIUD insertion techniques, and complications management; (2) hosted informational workshops for nurses and midwives at satellite clinics on PPFP counseling techniques; (3) provided PPFP leaflets to be distributed during counseling; (4) provided a video to be played in hospital waiting areas; (5) supplied Kelly forceps for vaginal PPIUD insertion; and (6) conducted regular monitoring and support.

### Provider Training

The PPIUD Initiative applied a “train-the-trainer” approach whereby a group of master trainers were identified in each country, trained providers on PPFP counseling and PPIUD insertion, and then those providers were expected to cascade training to other staff after returning to their hospital. Master trainers were accredited by the Tanzania Ministry of Health and Social Welfare. All providers eligible to insert IUDs were to receive training, and refresher trainings were to be conducted as needed. Three days of training were dedicated to counseling, and 3 days to insertion. During the 6-day training period, providers received a daily allowance of the equivalent to US$55 to cover the cost of lodging and meals.

During counseling training, trainers presented detailed information on all available methods including PPIUD. Providers were encouraged to share their opinions on PPIUD so that underlying prejudices could be addressed.^19^ Training sessions were interactive and included role-play with case scenarios of women with different family planning needs. Staff were encouraged to use learning aids (leaflets, posters, flipcharts, and videos) to enhance PPFP counseling sessions.

Providers at satellite clinics were trained on PPFP counseling only, but had the opportunity to observe and perform practice insertions on Mama-U models during their training. Those working in selected tertiary/teaching hospitals received additional off-site training on PPIUD insertion. Providers were trained to counsel women during routine ANC and at labor and delivery. Training covered the benefits and side effects of PPIUD and all other methods, as outlined in Tanzania’s national PPFP guidelines.^20^ Trainers also emphasized the voluntary nature of the program—that women have the option to opt in or out of PPIUD insertion both pre- and postpartum.

PPIUD insertion and removal training included both classroom-based theoretical training and practical sessions using the Mama-U postpartum uterus, an anatomical model intended for clinical training purposes. The technique taught used the long‐handled 33 cm curved Kelly forceps to ensure that the IUD was placed at the top of the fundus while the uterus is still enlarged as opposed to 24 cm tissue or sponge forceps, which do not reach the fundus and may lead to higher expulsion rates.^19^ Assessment of trainee competency following PPIUD insertion training was standardized across countries. To achieve competency, trainees had to successfully complete a minimum of 3 peer-assessed Mama-U insertions, 2 supervised live insertions, and 3 unsupervised live insertions.^19^

### Program Implementation

Women attending ANC at satellite clinics were intended to receive one-on-one PPFP counseling. In accordance with national PPFP guidelines around informed choice,^20^ counselors were expected to deliver information about all available family planning methods, including how they work, duration of use, effectiveness, and possible side effects. Women had the choice of method, and all methods were available free of cost. Available methods included condoms, oral contraceptive pills, emergency contraceptives, natural family planning methods, injectables, implants, IUDs, and voluntary surgical sterilization.^20^ Within the range of methods described, providers emphasized the advantages of PPIUD as a safe, effective, and long-acting method.^19^ They were also expected to demonstrate how the PPIUD is inserted using visual aids, brochures, and anatomical models. Pregnant women who received counseling during ANC visits had the option to provide advance consent to PPIUD insertion after delivery, and their medical charts were marked with their stated decision. Those who opted for PPIUD in advance were referred to a tertiary/teaching facility for delivery, where trained providers would insert the PPIUD. Women who consented during pregnancy were asked again at the time of delivery to confirm their choice of PPIUD insertion, at which point they could refuse PPIUD without consequence. Additional counseling on other family planning methods was available to all women. Women who received a PPIUD were advised to return for a follow-up visit at 6 weeks postpartum to ensure proper placement of the IUD. In some cases, IUD threads were trimmed at follow-up if women complained of feeling them or reported discomfort during intercourse.

Four levels of supervision and quality assurance were put into place. First, facility-level coordinators provided weekly supervision. Second, the AGOTA monitoring and evaluation team conducted quarterly supervision visits. As part of AGOTA’s routine monitoring, trained data collection officers conducted exit interviews with all consenting women after delivery and before discharge. These interviews captured data on whether women received PPFP counseling, and whether they consented to having PPIUD inserted. Third, the Tanzania Data and Safety Monitoring Board reviewed progress semiannually. Fourth, a steering committee comprised of experienced gynecologists and the national coordinators provided high-level oversight. Providers who had difficulties with their insertion technique received quarterly refresher trainings throughout the initiative.

Key achievements of the PPIUD Initiative in Tanzania are summarized in Table 2. A prospective cohort study nested within Tanzania’s PPIUD Initiative reported that 5.8% of women who delivered at project hospitals during the study period had a PPIUD inserted.^21^ Forty-three percent of women with a PPIUD returned to a project-affiliated clinic for follow-up visit 4–6 weeks after delivery. Among them, midwives performed 59% of PPIUD insertions, and clinicians performed 41%. PPIUD expulsion and removal rates were 1.2% and 8.3%, respectively.^21^

**TABLE 2.** PPIUD Initiative Achievements in Tanzania

**Achievements**	
Participating hospitals, n	6
Providers trained under the PPIUD Initiative, n	827
Women counseled on family planning and PPIUD, n	21,479
Counseled during antenatal care, %	57.0
Counseled only after admission for delivery, %	43.0
Deliveries during the PPIUD Initiative period, n	91,387
Women followed up for postpartum interview, n	80,194
Women who consented for PPIUD insertion, n	5,634
PPIUD insertions, n	3,095

Abbreviation: PPIUD, postpartum intrauterine device.

### Parent PPIUD Study

This qualitative investigation was nested within the Tanzania PPIUD parent study, which evaluated the causal effect of the initiative on the uptake and subsequent continued use of PPIUD in 3 countries: Nepal, Sri Lanka, and Tanzania. The study in Tanzania was conducted in tertiary/teaching hospitals in 5 cities: Arusha, Dodoma, Dar es Salaam, Mbeya, and Pwani. A hospital and 3–4 of its satellite clinics were selected in each location (see research protocol for detailed procedures).^9^

In the parent study, the PPIUD initiative had only a modest impact on women’s choice of PPIUD (an increase of 6.3 percentage points) due to low coverage of PPIUD counseling among women delivering in study facilities.^22^ Adherence-adjusted estimates suggest that if all women had been counseled as intended, we could expect a 31.6 percentage point increase in choice of PPIUD. Qualitative findings from the present study are intended to contextualize these results by highlighting strengths and weaknesses in program implementation and potential opportunities to improve future implementation of similar interventions.

Identifying factors that influence program implementation is essential for assessing the fidelity of interventions and understanding why they were or were not effective. For example, negative results can occur when interventions are not implemented sufficiently, and similarly, positive results can be achieved by an intervention that was delivered differently than intended.^23^ Therefore, understanding what contributes to implementation success or failure is critical to program improvement, replication, and scale-up across settings.

## METHODS

### Study Design and Data Collection Procedures

One objective of the evaluation was to understand how the initiative was implemented, the perspectives of providers on implementation, and receptiveness of women toward PPIUD services. In the present study, we conducted qualitative in-depth interviews (IDIs) with providers and women who participated in the initiative to assess the implementation barriers and facilitators.

The consolidated criteria for reporting qualitative research (COREQ) was used to ensure complete reporting of qualitative procedures (Supplement 1). Sample sizes for IDIs were estimated based on what would be sufficient to achieve saturation in themes and study aims.^24^ Authors recruited and trained 2 female Tanzanian interviewers, who were hired as independent consultants. The interviewers each had bachelor’s degrees in sociology and more than 10 years’ experience conducting qualitative interviews. Authors oriented the interviewers on study procedures including the ethical considerations, informed consent process, and interview guides, as well as oversaw piloting and data collection. The interviewers had no prior relationship to study participants, and participants had no prior knowledge of any research team members.

We conducted IDIs with 15 providers between June 2016 and February 2017, approximately 3 months after they received PPIUD training, to understand their experiences with the training and perceived facilitators and barriers to implementation. We purposively selected 2–4 providers from each hospital who had participated in the training and were actively delivering the initiative. Researchers contacted providers by phone to inform them about the interviews, briefly explain the purpose of the interviews, and request participation.

We conducted IDIs with 47 women exposed to the initiative to understand their experiences with PPFP counseling, and their decision making regarding postpartum contraceptive use. Between June 2016 and February 2017, we conducted IDIs with 20 pregnant women who had at least 2 ANC visits in a study facility, received PPFP counseling, and were offered PPIUD insertion. From each site, we purposively sampled 4 pregnant women to include a mix of women from higher- and lower-income levels and range of ages. Eligible women were initially approached by providers at the end of their ANC appointment to inform them about the study. Women who were interested to learn more were referred to the research team. Interviewers assessed women’s eligibility independent of providers, explained the purpose of the study, administered informed consent, and conducted private one-on-one interviews after an ANC visit.

Between April 2018 and August 2018, we interviewed a separate sample of 27 postpartum women who had received a PPIUD. The parent study collected data on whether women continued using the PPIUD, discontinued due to expulsion, or intentionally discontinued before the qualitative interviews. We aimed to recruit 10 women from each of these groups; however, the number of women who experienced expulsion was small (1.2% in the parent study), thus we were only able to recruit 7 women. These women were randomly selected for interviews from the parent study database, which included detailed contact information for enrolled women. Researchers contacted women by phone to inform them about the qualitative interviews, briefly explain the purpose of the interviews, and request participation.

We developed semistructured interview guides with open-ended questions in English and translated them into Swahili. The interview guide for providers covered the following topics: knowledge, experiences, and preferences for contraceptive methods; PPIUD training experience; and implementation, scale-up, and diffusion of PPIUD services. Interview guides for women included questions to assess contraceptive knowledge and prior use, as well as their experiences and perceptions of PPFP counseling and postpartum contraceptive decision making. For postpartum women who received a PPIUD, the guide included questions regarding their experience with the PPIUD Initiative, including content and perceived quality of PPFP counseling. We also asked questions about their experiences using the PPIUD, including reasons for continuation or discontinuation. Tanzanian researchers verified translations and back-translated the guides to ensure content and semantic equivalence of each question.^25^ We pretested interview guides to assess question phrasing, sequencing, and overall comprehension and modified the guides as appropriate.

Before each interview, participants were asked to provide written consent to take part in the study. Women who were unable to sign their names provided a thumbprint along with a witness’ signature. All providers gave a signature. One-on-one interviews were conducted in Swahili, and in a private space at the facilities or another private location if participants preferred. Interviewers made field notes during interviews that were used during transcription/translation to add contextual details. Interviews were audiorecorded with participants’ permission and subsequently transcribed and translated to English for analysis. Interviews lasted between 60 to 90 minutes. No one refused to participate in the study.

### Ethics Approval and Consent to Participate

Ethical approval as exempt was granted by the Institutional Review Board at Harvard University. The study received ethical approval from the National Health Research Ethics Review Committee of the National Institute of Medical Research in Tanzania. The consent statement included the purpose of the study, confidentiality of personal information, and the use of information for publication. Only those who consented were interviewed.

### Analytical Strategy

We used ATLAS.ti (Version 8.0) to manage, code, and interpret transcripts using thematic content analysis. We applied a multistage analytical strategy to identify key themes, codes, and subcodes.^26^ In the first stage, we prepared an initial list of parent codes and definitions based on study aims, interview guides, and existing literature on PPFP. Examples of parent codes include: counseling implementation, IUD, other family planning methods, decision making, and quality of services. We applied these high-level codes to group the data in our first pass through the transcripts.^26^ During this process, we identified relevant subcodes under each parent code. For example, under the “counseling implementation” code, we added the following subcodes: client receptiveness, counseling frequency, counseling content, counseling provider, and counseling timing. Under “decision making” we identified the following subcodes: support, trust, method choice influence, discontinuation, fertility limit, fertility space, fertility continue, coercion, and trade-offs. We developed a preliminary codebook, which included both predetermined and emergent codes. Next, we divided the transcripts between researchers and independently coded line-by-line using the codebook. We wrote analytical memos to summarize case details, highlighting particularly rich narratives and emergent themes. We reviewed each other’s coding and came to agreement on categories and themes to ensure analytical rigor and consistency across transcripts.^26^

In the final stage, we analyzed the coded transcripts using 2 theoretical frameworks (described later) to answer the following questions: (1) What were the barriers to, and facilitators of, PPIUD implementation? and (2) At what level did each of these barriers and facilitators operate? To do this, we sorted coded sections of the transcripts into “bins” that correspond to elements of each framework. This process was done manually in Microsoft Excel to enable visualization of the sorted data in a matrix. We then analyzed all the quotes within each element/level of the frameworks to further categorize them into barriers or facilitators.

#### Theoretical Frameworks

To gain insight into the successes and challenges of the PPIUD Initiative’s implementation, we applied the implementation outcomes framework developed by Proctor and colleagues.^27^ The framework differentiates between 3 “distinct but interrelated” sets of outcomes: implementation, service, and client outcomes. Implementation outcomes are defined as^27^:

*the effects of deliberate and purposive actions to implement new treatments, practices, and services*, … *which* … *serve as necessary preconditions for attaining subsequent desired changes in clinical or service outcomes downstream*.

**FIGURE 2.**
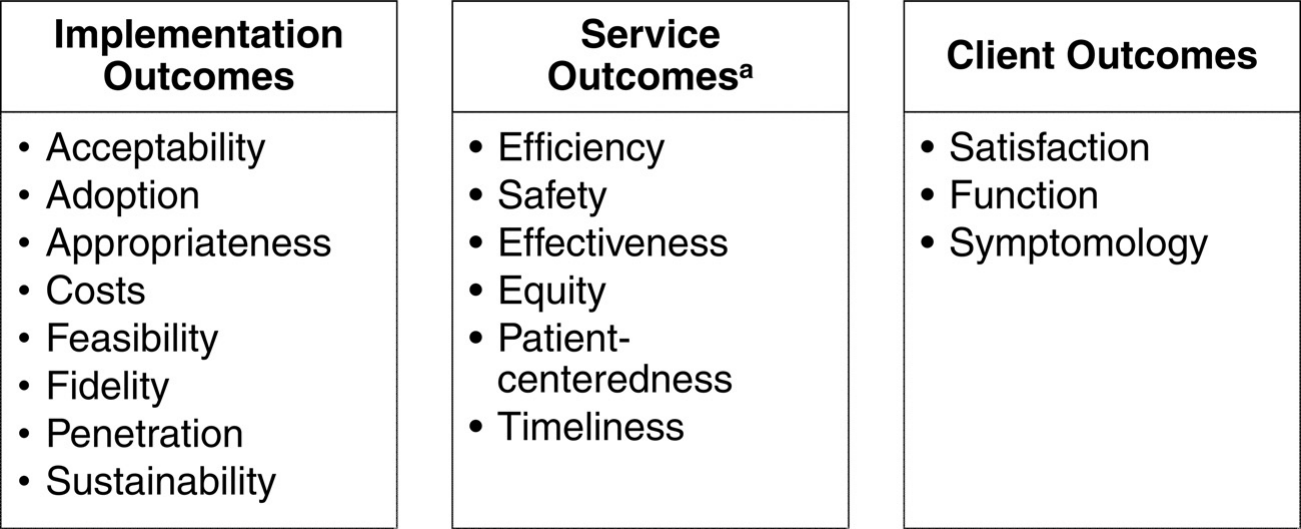
Visual Representation of the Implementation Outcomes Framework^27^ ^a^Institute of Medicine standards of care.

Figure 2 shows a visual representation of the framework developed by Proctor et al. Application of this framework helps advance our theoretical understanding of the implementation process of the PPIUD Initiative in Tanzania and evaluate the drivers of successful implementation using common nomenclature and a systematic analytical approach.

In conjunction with the implementation outcomes framework, we used an ecological framework adapted by the United States Agency for International Development’s Maternal and Child Health Integrated Program for PPFP,^28^ to categorize factors influencing implementation into 5 different levels: individual (woman), partner/family, health system, community, and policy. Implementation of novel interventions is intrinsically multifaceted, thus it is important to measure outcomes at different levels to understand areas of success and failure and potential opportunities for improvement. Yet, few studies specify the level and rarely address how different levels relate to one another.^27^ Thus, the ecological framework is a useful tool to guide our assessment of this PPFP initiative by highlighting the contextual, health system, and policy-level factors that influenced implementation.

## RESULTS

**FIGURE 3.**
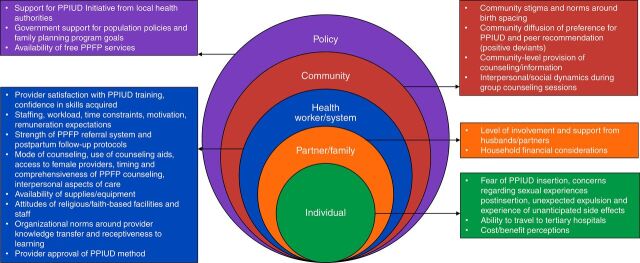
Ecological Framework Illustrating Factors That Influenced Implementation of the PPIUD Initiative in Tanzania Abbreviations: PPFP, postpartum family planning; PPIUD, postpartum intrauterine device.

Demographic characteristics of women and providers are summarized in Tables 3 and 4. Analyzing transcripts using the 2 frameworks described revealed a number of factors that influenced PPIUD implementation. In Figure 3, we summarize findings by level in the ecological framework and include these summaries in the descriptor boxes for each level. In the text that follows, we apply the Proctor framework to categorize these factors as barriers and facilitators under implementation outcomes, service outcomes, and client outcomes (Table 5).^27^ Illustrative quotes are included in the text, and additional quotes are in Supplement 2. We did not find any meaningful differences in women’s perceptions of how the intervention was implemented by age, sociodemographic status, or location.

**TABLE 3.** Number and Percent Distribution of Women, by Background Characteristics and Interview Type

	**Interviews With Pregnant Women**	**Interviews With Women Receiving PPIUD**
	**n (%)**	**n (%)**
**Geographical region**		
Mbeya	3 (15)	9 (33)
Arusha	6 (30)	5 (19)
Dodoma	2 (10)	4 (15)
Dar es Salaam	5 (25)	6 (22)
Pwani	4 (20)	3 (11)
**Age, years**		
<20	1 (5)	1 (4)
20–24	3 (15)	3 (11)
25–29	10 (50)	13 (48)
30–34	3 (15)	2 (7)
35–42	2 (10)	7 (26)
Missing	1 (5)	1 (4)
**Education**		
Some Primary	1 (5)	0 (0)
Completed Primary	3 (15)	6 (22)
Some Secondary	3 (15)	1 (4)
Completed Secondary	10 (50)	15 (56)
More than Secondary	2 (10)	4 (15)
Missing	1 (5)	1 (4)
**Marital Status**		
Married	15 (75)	21 (78)
Single, not living together	2 (10)	3 (11)
Single, living together	1 (5)	0 (0)
Widowed	0 (0)	2 (7)
Missing	2 (10)	1 (4)
**Occupation**		
Unemployed	5 (25)	6 (22)
Homemaker	1 (5)	0 (0)
Business owner	5 (25)	9 (33)
Teacher	2 (10)	4 (15)
Other (e.g., nurse, secretary, salonist)	5 (25)	7 (26)
Missing	2 (10)	1 (4)
**Religion**		
Christian	15 (75)	20 (74)
Muslim	3 (15)	6 (22)
Missing	2 (10)	1 (4)
**Total No. of Children (alive or deceased)**		
0	6 (30)	0 (0)
1	8 (40)	11 (41)
2	2 (10)	7 (26)
3 or more	3 (15)	8 (30)
Missing	1 (5)	1 (4)

Abbreviation: PPIUD, postpartum intrauterine device.

**TABLE 4.** Number and Percent Distribution of Providers by Background Characteristics

**Characteristic**	**n (%)**
Geographical Region	
Mbeya	4 (27)
Arusha	4 (27)
Dodoma	2 (13)
Dar es Salaam	2 (13)
Pwani	3 (20)
Gender	
Male	2 (13)
Female	13 (87)
Age	
29–39	7 (47)
40–50	4 (27)
>50	2 (13)
Missing	2 (13)
Profession	
Physician	3 (20)
Nurse	12 (80)
Length working in profession, years	
≥5	5 (33)
6–15	4 (27)
≥16	3 (20)
Missing	3 (20)
Length providing family planning services, years	
≥5	5 (33)
6–10	7 (47)
≥11	1 (7)
Missing	2 (13)

**TABLE 5.** Application of the Implementation Outcomes Framework to Assess Facilitators and Barriers to PPIUD Initiative Implementation in Tanzania

**Outcome and Definition**	**Facilitator**	**Barrier**
Implementation Outcomes		
Acceptability: Perception among stakeholders that intervention is acceptable (e.g., satisfaction with PPIUD training content, complexity, comfort)	High satisfaction with PPIUD training	Lack of providers trained on PPIUD insertion Lack of support from local health authorities
Adoption: Initial implementation of PPFP counseling and PPIUD insertion; Intention to try	Increased confidence following PPIUD training	Time constraints and inadequate staffing Gaps in referral system between satellite clinics and hospitals
Fidelity: Delivered counseling as intended (e.g., reach, content, and target population)		Individual counseling replaced by group counseling Diminished provider motivation Counseling rushed or skipped Skewed or incomplete counseling
Penetration: Diffusion of PPIUD Initiative within intervention facilities and to other non-intervention sites	Emphasis on PPIUD’s mechanism of pregnancy prevention during training	Objections from faith-based facilities Expectation for remuneration among staff who did not receive initial training
Sustainability: Long-term maintenance and institutionalization of the PPIUD Initiative	Support for population policies and family planning programs to achieve fertility reduction goals	Breakdown of supply chain and stock-outs
Service Outcomes		
Equity: Extent to which the PPIUD implementation is equally available/accessible to all intended beneficiaries		Differential treatment by health care providers Financial barriers to accessing hospitals Lack of community-based PPFP counseling and services
Client Outcomes		
Client receptiveness/demand for services: Client receptiveness to being counseled on PPFP and/or demand for receiving the PPIUD	Level of support from husband/partner Shared intention among couples to space pregnancy for financial reasons Community and gender norms related to birth spacing Community diffusion of preference for PPIUD and peer recommendation Women's trust in provider advice Cost-free counseling and insertion services	Fear of insertion, concerns related to sexual experiences postinsertion, unexpected expulsion and experience of unanticipated side effects (results published elsewhere^26^).
Satisfaction with PPIUD services: Client receptiveness to being counseled on PPFP and/or receiving PPIUD; Satisfaction with counseling and services	Delivery of counseling and services by female provider Interpersonal aspects of care	Perceived provider incompetence

Abbreviations: PPIUD, postpartum intrauterine device; PPFP, postpartum family planning.

### Implementation Outcomes

#### Acceptability

Acceptability refers to the perception among implementation stakeholders that an intervention is “agreeable, palatable, or satisfactory” and can be assessed qualitatively based on individuals’ opinions of the intervention’s content, complexity, or comfort.^27^ We identified a majority of these factors at the health system level.

**Facilitators.** The most important facilitator of intervention acceptability at the health system level was high satisfaction with the PPIUD training among providers. Providers were highly content with the training in terms of the content covered, complexity of information provided, and comfort in implementing the skills they learned. One participant articulated this particularly clearly, and most providers shared these views:

*Implementation was good … I can say motivation was high because of the way training was conducted. It equipped people with knowledge and each person came out feeling that she is capable of doing PPIUD insertion.*—Provider

**Barriers.** Both women and providers reported that too few staff were trained specifically on IUD insertion. The limited number of trained inserters was perceived as a missed opportunity, creating additional barriers for women seeking PPFP services. For example, some women consented to PPIUD insertion during ANC but failed to have the method inserted due to lack of available trained providers:

*More providers should be trained on PPIUD insertion … a woman that I advised to get the PPIUD - when she went to the hospital, the health provider who could provide the service was not around that day. Many women may be discouraged if they experience this. For a better service, we need providers that can give the services whenever needed.* —Woman, postpartum

One provider who was trained only on PPFP counseling expressed a desire for additional training on insertion.

A policy-level barrier to implementation was a lack of support from local health authorities. According to a provider, the district reproductive and child health coordinator imposed administrative hurdles to timely implementation, noting that it was difficult to convince the coordinator of the intervention’s value, which, in turn, delayed the process of obtaining the necessary equipment in time. This suggests that while acceptability was high among training recipients, it was lower among administrative authorities who played a key role in implementation.

#### Adoption

Adoption is defined as a provider’s “intention, initial decision, or action to try or employ” an intervention.^27^

**Facilitators**. Increased confidence among providers following the PPIUD training was a key facilitator of adoption. Although some providers felt the training could be longer, almost all claimed that they left the training with new knowledge. Many reported increased confidence in their ability to offer family planning counseling and education because of the training received:

*I was not competent with family planning [prior to the training], but I was able to learn about the other methods too because we were taught briefly [about all methods] during the training. I came out feeling that I am capable of caring for a woman and all challenges that may come up, and all the misconceptions related to IUD.* —Provider

**Barriers**. A commonly cited barrier to PPIUD counseling adoption was time constraints among already overburdened staff. Despite their satisfaction with training content, some counselors had insufficient time to implement what they had learned.

Time constraints were compounded by shortages in facility staff, and as a result, PPFP counseling was often deprioritized or adaptions were made to ease provider workload. Time constraints were particularly problematic when women received PPFP counseling at the time of hospital admission before delivery, which may have led to rushed and/or incomplete counseling. To cope with time constraints at the time of delivery, a provider described a process of triaging patients for PPFP counseling based on previous exposure to information. If a woman received PPFP counseling during pregnancy, then providers streamlined the topics discussed:

*Due to time limitations, you ask a woman if she has ever received any counseling previously. If yes, then you only focus on the key points because we know she has received information from the antenatal clinic. So we emphasize the advantages of birth control, minor side effects and their symptoms.* —Provider

Providers also described gaps in the referral system between satellite clinics and larger referral hospitals. Within this system, PPIUD insertion providers relied on satellite clinic nurses to provide high quality PPFP counseling, make appropriate referrals, and provide timely follow-up care, which was not always implemented as intended:

*We often say tragedies that occur in the referral hospitals are caused by facilities at the lower level. … This is due to the nurse’s carelessness, perhaps not doing their job well. If people don’t get proper counseling at the clinic, we will end up having problems here (at the hospital).* —Provider

Although the PPIUD referral system was intended to streamline project implementation, several hospital-based providers felt there was limited oversight of providers in smaller clinics. One provider was particularly concerned about 6-week follow-up procedures, in which women were advised to return to the hospital to have the IUD placement checked and threads shortened if necessary. According to a provider, if a client returned to a facility that was unable to provide quality follow-up care, then she may have had a negative experience and discouraged others from adopting PPIUD.

#### Fidelity

Fidelity is the degree to which an intervention was implemented as initially intended by design. Providers described several adaptations to the intended PPIUD intervention, primarily in response to barriers related to adoption.

**Barriers**. To cope with time and staff shortages, providers invited pregnant women to attend scheduled group PPFP counseling sessions rather than the intended one-on-one counseling during ANC visits. Shifting to group counseling helped to reduce the burden on busy providers, but social dynamics between older and younger women may have influenced some women’s willingness to fully participate:

*Although counseling women as a group is good, I think there is also a need to have individual sessions where a woman can ask [her own] questions. Some women, especially those who are old like me, may fail to ask questions when they are in a group of young women, some of whom are teenagers.* —Woman, postpartum

Although providers considered group family planning counseling to be an effective time management strategy, they also acknowledged that it was difficult for some women to attend at the specified times due to competing household demands. Since it was not feasible for providers to deliver comprehensive one-on-one counseling throughout the day, women who were unable to attend group sessions or who arrived late would miss counseling completely; a deviation from the intended intervention. This challenge was linked to diminished motivation, rushed counseling, and/or missed counseling opportunities:

*The nurses are overwhelmed and tired. There are days that you go to the clinic to get services, but you leave without getting educated or counseled on anything. Yet, when women gather at the clinic that is the best platform to explain about the methods for family planning … When the woman goes back home, she will appreciate that she has learned something and when she delivers, she will have already decided on which family planning method to use.—*Woman, postpartum

Lastly, counselors were trained to counsel on all available methods to enable informed choice and ensure that women understand the full range of PPFP options, including their benefits and potential side effects. However, women’s narratives demonstrated that PPFP counseling was highly skewed toward PPIUD, and some may have received incomplete information (or could only recall partial information) on the possible physical side effects of the PPIUD. This suggests a lack of fidelity to the training delivered.

*[The nurses] told us it works for 10 to 12 years’ time and can be inserted just after delivery … and you may remove it at any time. And this method has no side effects unlike implants, which may cause long term bleeding or lack of menstruation at all. But these new methods, you will still have your menstrual cycle as usual and have no side effects. Unpleasant effects can occur for the first 3 months but not longer.* –Woman, prenatal

#### Penetration

Proctor et al. define penetration as the “integration of a practice within a service setting and its subsystems.”^27^ Penetration is closely related to the concept of “diffusion” and is typically measured quantitatively; however, qualitative analysis revealed important insights into how the initiative could be better integrated into existing service delivery environments. Here, we consider penetration to include diffusion of the initiative both within participating intervention facilities and to other local facilities not selected for the PPIUD Initiative.

**Facilitators**. When service providers reflected on the potential for diffusion of the initiative to other facilities, they identified a number of factors operating at the health system level. One provider explained that receiving focused training on the PPIUD’s mechanism of action clarified their misconceptions about how PPIUD functions and relieved moral concerns about providing the method:

*I heard from Catholics that using loops is killing children and you are killing every month, so that thinking affected me … Well according to the training it is not true … [Prior to PPIUD training] when I was advising the woman about the methods, afterwards I regretted that, thinking, “my God, I might have killed” … I personally don’t have any obstacles now, even if I go back to the Roman Catholic [facility] where I studied, I will educate them about the [PPIUD].*––Provider

**Barriers**. The most common potential barrier to diffusion was that faith-based facilities would object to implementing PPIUD services. Consequently, both women and providers believed that a PPIUD intervention would only be effective and sustainable in government facilities. Another health system-level barrier was the expectation for remuneration among other staff who did not attend the off-site training. Providers explained that diffusion of information and learnings to other colleagues could be a challenge because PPIUD training participants received an allowance. Consequently, other staff may perceive this arrangement to be unfair. Despite this challenge, some staff were reportedly open to learning, without expectations of rewards. This was dependent on the workplace culture of specific facilities:

*There are some who received it well. At this facility, we have a norm that if you go to a training, when you come back you have to provide feedback. So when you’re on shift and you have some time, you can instruct and teach others what you learned […] but not all staff do this because some may complain, saying, “You got the money and now you come back with just words.”* ––Provider

#### Sustainability

Intervention sustainability is the extent to which a newly implemented intervention is “maintained or institutionalized within a service setting’s ongoing, stable operations.”^27^ Penetration and sustainability are conceptually similar, but temporally distinct, as higher penetration typically contributes to long-term sustainability.

**Facilitators**. Most health care providers believed there was strong political will among government officials to invest in strategies that would achieve fertility reduction goals. This was viewed as a policy-level facilitator of PPIUD sustainability beyond the life of the project:

*I don’t see any reason why it would fail because we have a problem of high population, and we have an intervention that can reduce this population growth rate, so why can’t the government support this? … If they are able to supply other services then why not this as well … it’s a national priority, and it is in the sustainable development goals!* ––Provider

**Barriers.** A key driver of successful institutionalization at the health system level was having the necessary instruments and equipment readily stocked and available, which requires support from and coordination with Ministry of Health authorities. At the time of the study, AGOTA supplied PPIUD equipment at no charge. Many respondents expressed frustration with the shortage of PPIUD equipment and supplies, and, as more women learned about the PPIUD Initiative, equipment shortages became increasingly problematic. Despite an increased influx of patients, there was no equivalent increase in equipment and supplies to meet the heightened demand. Providers felt strongly that even if other government facilities agreed to provide PPIUD services, the inconsistent provision of supplies would be problematic. This raised questions about the long-term sustainability of the intervention without AGOTA support:

*To be honest, it will be difficult to supply the instruments to other facilities… that is not easy, and it may take a long time because for now, our facility is supplied by donors. That’s why it’s possible. But with the government, you may write a request for the material until all the ink in that pen is gone, and still not get what you asked for!* ––Provider

### Service Outcomes

#### Equity

**Barriers**. Equity was the main service outcome construct to emerge from IDIs. At the health system level, a key barrier to equitable PPFP counseling was differential treatment by health care providers. One woman suggested that adolescents are reluctant to seek family planning counseling out of fear of judgment by health care providers. In addition, some providers reportedly prioritized women who attended antenatal clinics with their husbands, whereas those who attended alone waited longer for services:

*They would prioritize women who came with their husbands. It did not matter whether you were there first, they would ask those who came with their husbands to go in for the services first […] In most cases, my husband is always with me because he knows when you go to the clinic with a man, you will be given priority.* ––Woman, postpartum

These reports indicate a lack of fidelity to the intervention, as providers were trained to counsel *all* ANC clients and to tailor contraceptive method recommendations according to women’s individual needs regardless of age or marital status.

Another barrier to equitable service delivery was the cost of accessing large hospitals. One provider stated that lower income women have a tendency to deliver at peripheral facilities because they are less busy, closer to home, and less costly to access. Transportation to large hospitals for delivery or PPIUD services may not be feasible for women with fewer resources:

*Another challenge is women’s economic status. Some give birth here (at the health center) for free, but when you ask her to take a [USD$0.86] bajaji (motorized rickshaw) to [the hospital], she can’t manage it, so we feel that we are going to lose them … and the providers there (at the hospital) are overloaded, so women are scared. They prefer to get the delivery services here … the hospital is very busy.* ––Provider

Lastly, although the PPIUD Initiative was designed for implementation only at the facility level, both women and providers viewed the lack of community-based PPFP counseling as a missed opportunity for equitable service delivery:

*We should also get out of this hospital and educate people in the village because problems are not only in town. A majority who face challenges are in the villages, in our districts … we have to go there and train people.* ––Provider

### Client Outcomes

#### Client Receptiveness and Demand for Services

Although women’s receptiveness was not included in the original implementation outcomes framework, we consider it an important addition, as factors motivating or discouraging engagement with the intervention may directly influence both implementation and PPIUD uptake.

**Facilitators**. Women identified several facilitators of PPIUD uptake at the partner/family level. In addition to feeling supported by health care providers, several women highlighted the important role of their husbands in supporting their decision to use PPIUD. Another motivator to use PPIUD was a shared intention among couples to space pregnancy for financial reasons:

*Depending on my business and the way I planned with my husband, I know that if I use the loop, there are some things we will be able to accomplish before getting pregnant again.* ––Woman

At the community level, 2 important factors facilitated women’s receptiveness to PPFP counseling and PPIUD services. The first was community norms around preference for long birth spacing. When asked to explain why she felt it was important to prevent pregnancy soon after birth, a woman described the shame and stigma associated with short birth spacing:

*First, it’s because the baby will still be very young, [and] second, I feel shame when I am in the community, getting pregnant when the baby is only about 4 or 5 months old; it’s a shameful thing! […] If you happen to get pregnant [again], they tend to put all the weight on women, like, “how did you let yourself get pregnant?” He won’t abandon you, but he will put all the blame on you, saying that you did it on purpose.* ––Woman, prenatal

Second, the presence of women who had positive experiences using PPIUD was a critical community-level facilitator of PPIUD counseling and service uptake. Participants described a community diffusion effect in which nonusers were encouraged by “positive deviants”––peers who had opted for the PPIUD and could speak from firsthand experience:

*At some point I was acting like an ambassador for the IUD because many women would come to me; they felt that I understood more. Even the health service providers would tell women to [talk to me] and I would share with them what the IUD was all about. Many women questioned how the PPIUD could be inserted immediately after delivery. They thought it was impossible.* ––Woman, postpartum

Despite the strong influence of their peers and neighbors, women were generally receptive to biomedical information and the advice provided by medical professionals, and most were likely to show interest in the PPIUD if providers demonstrated their own approval of the method.

At the policy level, the most important facilitator of PPIUD uptake was the availability of cost-free insertion services. Despite Tanzania’s policy to provide free health services for pregnant women and users of family planning services, some women were not aware that PPIUD and other contraceptive methods were available free of cost. As a provider noted, informing women of this policy influenced their decision to choose the method.

**Barriers**. We identified several important barriers to PPIUD demand at multiple levels. Many individual-level factors influenced women’s decisions to use PPIUD, which we have reported elsewhere in depth.^29^ These included fear of insertion, concerns related to sexual experiences postinsertion, unexpected expulsion, and experience of unanticipated side effects.

#### Satisfaction

**Facilitators**. At the health system level, women’s PPFP counseling experience influenced their overall satisfaction with PPFP services. These included the use of counseling aids during prenatal counseling (leaflets, waiting room videos, and brochures), and most importantly, having access to female family planning service providers. Women were generally more satisfied with care and more motivated to use PPIUD when counseled by female nurses who could speak to their personal experience using family planning. Women also appreciated providers who were respectful, compassionate, and patient, indicating that interpersonal aspects of care are a valued component of PPFP services:

*I am very grateful to them [providers] because they gave me all the support I needed. They were ready to answer my questions even before I started using the PPIUD, when I had it and when I went to remove it … They have really been supportive, even after removing the PPIUD … calling to ask if I was doing fine and if I needed any assistance with family planning methods.* ––Woman, postpartum

**Barriers**. Although most providers reported high confidence in their technical skills after training, some women reported contrasting perceptions of providers’ clinical skills. Several women perceived technical incompetence as a primary contributor to adverse PPIUD outcomes, including expulsion and/or improper placement, and warned that such negative experiences might deter future patients from using PPIUD:

*The person that inserted the PPIUD for me did not insert it well. She struggled very much, and it was like a trial and error thing … It was very painful because I had just given birth. When I got home, the PPIUD was expelled … The providers giving PPIUD services should be trained [on insertion] and should only be allowed to provide that service when they are competent; otherwise, many women run away from using the IUD.* ––Woman, postpartum

## DISCUSSION

This study applied 2 theory-based frameworks to assess the implementation of a postpartum contraception intervention in 5 Tanzanian hospitals. Applying a PPFP ecological framework to analyze qualitative IDIs demonstrated that successful implementation and uptake of PPIUD counseling and services depends on a complex interplay of factors operating at multiple levels, spanning from the individual (woman) level to the policy level. In addition, using the implementation outcomes framework informs our theoretical understanding of the implementation process, illuminating the “black box” of implementation dynamics, and highlights potential entry points for improvement in program delivery.^27^

Successful implementation of PPIUD counseling and services depends on a complex interplay of factors operating from the individual to the policy level.

Overall, acceptability of the PPIUD Initiative was high. However, providers perceived the selective training of some staff only on PPFP counseling, and others on both PPFP counseling and PPIUD insertion to be inequitable, which may have led to demotivation. The extent to which clinic staff are both “intrinsically” and “externally” motivated, valued, and supported in their professional roles directly influences program implementation.^30^^,^^31^ Previous research on promotion of IUD uptake in LMICs found that provider enthusiasm is a critical driver of implementation success.^18^ As such, providing remuneration only to providers participating in the off-site AGOTA-hosted trainings may have weakened possible opportunities of diffusion to other facility staff. AGOTA and FIGO believed the payments were justified given that some providers worked additional hours to support the initiative; however, this may have disincentivized employees who routinely work additional hours for other tasks.^19^ Further, ongoing payment is not sustainable,^19^ especially given that this intervention was implemented in a low-resource context.

There were many health system-level barriers to optimal implementation, including supply chain issues, gaps in referral between satellite clinics and tertiary/teaching hospitals, inadequate staffing and supplies, and overworked providers. A common narrative to emerge from both providers and women is that the existing health system infrastructure is fragile and any additional demands place further burden on providers. Consequently, the potential of any facility-based PPFP intervention is limited if existing health systems shortfalls are not addressed first. These findings are consistent with previous studies in Tanzania, which report irregularities in staffing, supplies and equipment, work overload, and communication challenges between facilities as barriers to implementation of maternal and newborn care.^16^^,^^30^^,^^32^^–^^34^

Both providers and women had similar views that the existing health system infrastructure is fragile and additional demands further burden providers.

Further, although there was high acceptance of the initiative, and providers repeatedly echoed the importance of PPFP on women’s health and well-being, they also perceived contraceptive counseling as a time-intensive endeavor, particularly when added onto routine ANC protocols. The perceived characteristics of an intervention can drive the adoption process, mediating the influence of intention to implement the program and actual behaviors to do so.^35^ A comprehensive needs assessment to evaluate feasibility and identify potential adaptations for the local context is recommended. For example, an audit of satellite clinics to assess providers’ current duties, workload, and willingness to take on additional tasks may have helped to identify clinics with more capacity to implement new initiatives.

Paradoxically, if an intervention leads to increased demand for services (as providers reported for PPIUD), this may diminish the quality of care provided in the long run if strategies to facilitate the increased workload are not in place. Another study in Tanzania found that unpredictable fluxes in uptake of maternal and newborn care created challenges for service delivery and directly influenced the quality of care provided.^16^ Although streamlining health care provision by integrating services (ANC and PPFP counseling, for example) is often promoted to improve health system efficiencies,^36^^,^^37^ such integration must be matched with proportional increases in staffing, training, and supplies. A scoping review of integration of HIV and sexual and reproductive health services similarly cautions that integration of maternal and reproductive health services must be judiciously planned in relation to current health systems functions.^38^

Integration of postpartum family planning services with existing services must be matched with proportional increases in staffing, training, and supplies.

Providers reportedly adapted to health system constraints by implementing scheduled group-based PPIUD counseling sessions for women attending ANC clinics. Their intention was to improve efficiency and reduce the burden of additional one-on-one counseling (as intended by the PPIUD Initiative). Although group-based PPFP counseling was a deviation from the intervention design, this modality has been successfully implemented in LMICs for other reproductive health issues,^39^^–^^42^ and may be warranted, particularly in resource-constrained settings. However, several providers indicated that in practice, some women were unable to attend the early morning group sessions and missed the opportunity for prenatal PPFP counseling. Additionally, some women had reservations about discussing PPFP in a group setting due to age-related social dynamics. Given that women seeking ANC are not a homogenous group, implementation of the PPIUD Initiative may have been strengthened if group sessions were stratified by age, parity, marital status, or education.

Group- versus individual-based PPFP counseling may produce different results, and the mode of counseling delivery likely influences the effectiveness of PPFP programs. Research from Northern Tanzania found that while PPFP counseling delivered alongside routine prenatal HIV-testing had an effect on postpartum contraceptive intentions, intentions were poor predictors of postpartum reproductive behavior.^43^ Taken together, results call for additional investigation of various integration models to determine the optimal timing and mode of PPFP counseling. Implementers might consider supplementing one-on-one PPFP counseling with group education opportunities to ease the burden on providers. However, these groups should be designed with social dynamics in mind to ensure that women feel comfortable to speak freely.

The PPIUD Initiative intended to serve women of all ages and socioeconomic status, and providers were expected to deliver comprehensive PPFP counseling covering all available methods. However, findings suggest that interpersonal aspects of care varied, with some women reporting rushed or incomplete counseling or an emphasis on the PPIUD over other methods. Further, the perception that some providers treated older women and/or those accompanied by their husbands more favorably during ANC suggests that fidelity to the intended PPIUD Initiative was not uniformly achieved. This finding aligns with a previous study in Tanzania in which adolescent mothers felt stigmatized by health care providers during ANC due to early pregnancy and childbearing.^44^ Such practices may reflect widely held sociocultural norms, and/or broader efforts to encourage male involvement in reproductive health,^45^ but may lead to unintended consequences in the long run. If unmarried and adolescent women (who are more likely to have unmet need for contraception) perceive differential treatment by health care providers, they may be discouraged from seeking future care. Women are also likely to discuss these experiences with peers, and a general mistrust of providers and/or reluctance to seek care may diffuse among communities. Unless equitable, high-quality care is delivered, these social diffusion dynamics will ultimately threaten the long-term sustainability of PPFP interventions.

Narratives suggest that women in our study were strongly influenced by their peers. One woman viewed herself as an “ambassador for the IUD” and took great pride in sharing her experience with others. Future initiatives could consider engaging women with positive experiences to help facilitate diffusion of information to their peers, both within the community and at facilities. This may be particularly useful in facilities where providers are overburdened.

Future initiatives could engage women with positive experiences to help facilitate diffusion of information to their peers.

Although women’s receptiveness to PPIUD services was strongly influenced by the experience of their peers, the perspectives of female providers were highly valued, primarily due to their ability to empathize with clients. This represents an important opportunity for intervention. However, women’s perceptions regarding differential treatment have concerning implications for long-term sustainability. Additional training and supervision to improve interpersonal aspects of care, including an emphasis on patient-centered counseling, informed choice, and respectful and nondiscriminatory service delivery should be integrated into future PPFP initiatives.

Narratives suggest that it is less feasible for women of lower socioeconomic status to deliver in tertiary teaching hospitals, despite referral to these facilities for PPIUD. Both delivery and PPIUD services were free of cost, but some women expressed concerns about distance, cost of transport, unfamiliarity with staff, and overcrowding in hospitals. This suggests that the PPIUD Initiative may unintentionally give privilege to women with greater financial means and those who may be more motivated to complete the PPIUD referral and deliver at tertiary hospitals. Although the initiative’s referral system was intended to streamline service delivery, the intervention does not address the barriers women encounter when attempting to complete referrals.

Additionally, the designated trainer at each teaching hospital was expected to provide cascade training and ongoing support to other staff; however, we do not have evidence to suggest that supervision strategies were implemented in the initial months to ease adoption or facilitate sustainability. Improved supervision strategies such as regular evaluation and feedback, strengthened organizational culture, and an emphasis on collaboration and partnership between facilities may improve implementation outcomes.^46^ To strengthen coordination between satellite facilities and hospitals, we recommend a stronger interfacility performance and quality improvement system be implemented from the outset of future initiatives.

### Strengths and Limitations

This was a qualitative study with purposively selected participants; therefore our results are not transferrable beyond the study sample. Interviewing both women and providers captured a range of perspectives on implementation, service, and client outcomes. However, interviews were often held at the participating facility, increasing the risk of social desirability bias. To minimize this risk, interviews were held in rooms with both audio and visual privacy. Given that many participants’ spoke candidly about their experiences and concerns, we believe social desirability bias was minimal. A further limitation is that we interviewed providers at only a single time point. Implementation outcomes are dynamic, and provider perspectives may change throughout the course of the initiative.^27^ However, implementation tends to be most difficult during its early stages, and by capturing providers’ immediate reactions and perceptions during the initial phase, we were able to highlight key opportunities for improved implementation of future PPFP initiatives.

## CONCLUSIONS

Renewed interest in postpartum family planning has ushered in a wave of interventions aimed to increase contraceptive use immediately following birth, including a focus on long-acting reversible methods. However, in LMICs such as Tanzania, health systems are overburdened, and providers often have limited resources to implement new initiatives with high fidelity. Constraints that impede implementation of novel PPFP programs in resource-poor contexts are often overlooked^46^; yet, given rapid development and eagerness to adopt effective programs, implementation strategies in these settings require attention.

Meeting women’s contraceptive needs is crucial to reduce adverse maternal and infant health outcomes in LMICs, yet there is limited research on the implementation of postpartum family planning programs in these contexts. Continued efforts to integrate contraceptive counseling into ANC as a part of national guidelines, and offering PPIUD insertion as part of a country’s method mix make implementation studies of such initiatives increasingly important.

## Supplementary Material

19-00365-Hackett-Supplement_2.pdf

19-00365-Hackett-Supplement_1.pdf

## Notes

**Peer Reviewed**

**Funding:** This study was funded by an anonymous donor.
