# Supplementary material for: Evaluating the Implementation of an Intervention to Improve Postpartum Contraception in Tanzania: A Qualitative Study of Provider and Client Perspectives
Source: Glob Health Sci Pract. 2020 Jun 30;8(2):270–89. doi: 10.9745/GHSP-D-19-00365 (PMC7326523; doi:10.9745/GHSP-D-19-00365)
Supplement: 19-00365-Hackett-Supplement_2.pdf [file 19-00365-Hackett-Supplement_2.pdf]

Supplement to: Hackett K, Huber-Krum S, Francis JM, et al. Evaluating the implementation of an intervention to improve postpartum contraception in Tanzania: A qualitative study of provider and client perspectives. *Glob Health Sci Pract*. 2020;8(2). https://doi.org/10.9745/GHSP-D-19-00365

Supplement 2. Application of the Implementation Outcomes Framework to Assess Facilitators and Barriers to PPIUD Initiative Implementation

**Outcome**	**Definition**	**Barrier or Facilitator**	**Barrier/Facilitator Identified and Illustrative Quotes**
**Implementation Outcomes**			
Acceptability	Perception among stakeholders that intervention is acceptable (e.g., satisfaction with PPIUD training content, complexity, comfort)	Facilitator	• **High satisfaction with PPIUD training**<br>*Implementation was good because after attending the training, ... within a short time we inserted over one hundred women… I can say motivation was high because of the way training was conducted. It equipped people with knowledge and each person came out feeling that she is capable of doing PPIUD insertion.*<br><br>*The important topics were how to receive [greet] a mother, her education, how you provide it, how you welcome her, explain to her the important contraceptive methods, their quality. The technique of letting the woman ask questions is the most important thing, I will counsel her but she has a chance [to speak] so that she can ask questions and I can measure her understanding.*<br><br>*The trainers gave us good education, we understood and managed to provide counseling to our clients, so I felt that we have acquired something and got the light to educate others. We were given working tools, for example stickers and pictures to show women during counseling, when we say where do we insert IUD to the woman, we demonstrate that using cervix picture, how the sperm appears… so you educate her with something that you can demonstrate - with that we feel that we have acquired something new.* —Provider

Supplement to: Hackett K, Huber-Krum S, Francis JM, et al. Evaluating the implementation of an intervention to improve postpartum contraception in Tanzania: A qualitative study of provider and client perspectives. *Glob Health Sci Pract*. 2020;8(2). https://doi.org/10.9745/GHSP-D-19-00365

Outcome	Definition	Barrier or Facilitator	Barrier/Facilitator Identified and Illustrative Quotes
		Barriers	• **Lack of providers trained on PPIUD insertion**<br>*Training should be increased because the counselors are many… we need more providers trained on insertion because currently we have twenty-four providers for insertion and we have more than one hundred and sixty people. Do you see how small the ratio is? ... If people who attended the training are not on a shift, the woman will miss the opportunity for PPIUD, so we need enough counseling and insertion trainings, they are all important.* —Provider<br><br>*More providers should be trained on PPIUD insertion because for example, a woman that I advised to get the PPIUD - when she went to the hospital, she was told that the health provider who could provide the service was not around that day. Many women may be discouraged if they experience this. For a better service, we need providers that can give the services whenever needed.* —Woman, postpartum<br><br>*I was satisfied with the training ... but I was not pleased with the fact that we were taught how to do counseling but not how to do the insertion. I really wish I was taught about insertion ... I was not trained for that, my [only] instrument is my mouth.* —Provider<br>• **Lack of support from local health authorities**<br>*Things which are needed to support [project implementation] ... well first is the cooperation from the DRCHCo. I heard that he went to the training a few days ago, but before that he was not accepting [the intervention]. Initially he responded that the letter [to request insertion equipment] was incomplete, and the chance vanished, [otherwise] we would have the insertors by now ... We feel that was a weakness because we depend on him as a coordinator* —Provider

Supplement to: Hackett K, Huber-Krum S, Francis JM, et al. Evaluating the implementation of an intervention to improve postpartum contraception in Tanzania: A qualitative study of provider and client perspectives. *Glob Health Sci Pract*. 2020;8(2). https://doi.org/10.9745/GHSP-D-19-00365

Outcome	Definition	Barrier or Facilitator	Barrier/Facilitator Identified and Illustrative Quotes
Adoption	Initial implementation of PPFP counseling and PPIUD insertion; Intention to try	Facilitators	• **Increased confidence following PPIUD training**<br>*I feel very grateful to receive this training. It is very important to me, and it has opened my mind. I was unaware about some things, I misinterpreted them, and I was thinking differently about others, so it has helped me. … My performance has changed; they have empowered me. They gave me knowledge, that's why things have changed, because if I was not aware I wouldn't have done anything.* —Provider<br><br>*I was not competent with family planning [prior to the training], but at least by starting with PPIUD I was able to learn about the other methods too because we were taught briefly [about all methods] during the training. I came out knowing that I am capable of caring for a woman and all challenges that may come up, and all the misconceptions related to IUD* —Provider

Supplement to: Hackett K, Huber-Krum S, Francis JM, et al. Evaluating the implementation of an intervention to improve postpartum contraception in Tanzania: A qualitative study of provider and client perspectives. *Glob Health Sci Pract*. 2020;8(2). https://doi.org/10.9745/GHSP-D-19-00365

Outcome	Definition	Barrier or Facilitator	Barrier/Facilitator Identified and Illustrative Quotes
		Barriers	• **Time constraints and inadequate staffing**<br>*It becomes so tiresome as you talk too much… in a single day we meet up to 40 women so it is very tough to talk to everyone with the same efficiency. For some you will be tired and you will be explaining things so quickly.* —Provider<br><br>*You are only three nurses with eighty patients, and eight pregnant mothers. You have to weigh them; you should know that the counseling that is provided is not like that of ten women together (in a group counseling session).* —Provider<br><br>*Due to time limitations you ask a woman if she has ever received any counseling previously. If yes, then you only focus on the key points because we know she has received other information from the antenatal clinic. So we emphasize the advantages of birth control, minor side effects and their symptoms ... Another thing is when you receive a client, you have to talk to her before she gets to the ward because you may not get time later.* —Provider<br><br>• **Gaps in referral system between satellite clinics and hospitals**<br>*We often say tragedies that occur in the referral hospitals are caused by facilities at the lower level … This is due to the nurse's carelessness, perhaps not doing their job well… like counseling - if people don't get proper counseling at the clinic, we will end up having problems here (at the hospital).*—Provider<br><br>*It is not mandatory for women [who have had PPIUD inserted] to return to us after six weeks; they can go to have the threads cut in other (satellite) facilities. If they experience difficulties at this stage, they might start spreading poison that if you insert PPIUD you will experience problems even when you reach the health facility, the providers don't want to help you.* —Provider

Supplement to: Hackett K, Huber-Krum S, Francis JM, et al. Evaluating the implementation of an intervention to improve postpartum contraception in Tanzania: A qualitative study of provider and client perspectives. *Glob Health Sci Pract*. 2020;8(2). https://doi.org/10.9745/GHSP-D-19-00365

Fidelity	Delivered counseling as intended (e.g., reach, content, and target population)	Barriers	• **Individual counseling replaced by group counseling**<br>*Although counseling women as a group is good, I think there is also need to have individual sessions where a woman can be given chance to ask [her own] questions. You know, some women, especially those who are old like me, may fail to ask questions when they are in a group of young women - some of whom are teenagers.* —Woman, postpartum<br><br>• **Diminished provider motivation**<br>*[You have to find] the motivation, because we are doing a lot of counseling, apart from our work ... It requires extra time - instead of leaving at three pm you might leave at four or five pm because you have to talk to every woman briefly. Despite the group health education, you still have to talk with [each] individual person.* —Provider<br><br>• **Counseling rushed or skipped**<br>*The nurses are overwhelmed and tired. There are days that you go to the clinic to get services but you leave without getting educated or counseled on anything. Yet, when women gather at the clinic that is the best platform to explain about the methods for FP… When the woman goes back home, she will be appreciating that she has learned something and when she delivers, she will have already decided on which FP method to use.* —Woman, postpartum<br><br>• **Skewed or incomplete counseling**<br>*[The nurses] told us it works for ten to twelve years' time and it can be inserted just after delivery… and you may also remove it at any time that you find convenient. And this method has no side effects unlike other methods. Unlike implants, which may cause long term bleeding or lack of menstrual bleeding at all. But these new methods, you will still have your menstrual cycle as usual and have no side effects. Unpleasant effects can occur for the first three months period but not longer.* —Woman, prenatal<br><br>*I have been told that I will still have my normal menstrual period as usual [while using PPIUD, and it has no side effects, unlike IUD. [IUD] may be placed in the uterus but you may still feel the pain from time to time, but this new method won't cause any pain or have any side effects.* —Woman, prenatal

Supplement to: Hackett K, Huber-Krum S, Francis JM, et al. Evaluating the implementation of an intervention to improve postpartum contraception in Tanzania: A qualitative study of provider and client perspectives. *Glob Health Sci Pract*. 2020;8(2). https://doi.org/10.9745/GHSP-D-19-00365

Outcome	Definition	Barrier or Facilitator	Barrier/Facilitator Identified and Illustrative Quotes
			I have decided to use this [PPIUD] because I have seen that it has no side effects, and they have said you can remove this, even at home… which is different from the implants. Although, I know I will not be able to remove it myself… I will come back here. —Woman, prenatal
Penetration	Diffusion of PPIUD initiative within intervention facilities and to other non-intervention sites	Facilitators	• **Emphasis on PPIUD's mechanism of pregnancy prevention during training**<br>*I heard from Catholics that using loops is killing children and you are killing every month, so that thinking affected me ... Well according to the training it is not true ... the PPIUD immediately prevents the sperm [and egg] from uniting, and they cannot continue to grow and form anything. [Prior to PPIUD training] when I was advising the woman about the methods, afterwards I regretted that, thinking 'my God, I might have killed' ... I personally don't have any obstacles now, even if I go back to the Roman Catholic [facility] where I studied, I will educate them about the [PPIUD].* —Provider
		Barriers	• **Objections from faith-based facilities**<br>*Perhaps if it's a religious hospital… I hear they don't provide family planning education. People learn outside the facility, so that may hinder the services from being available there.* —Provider<br><br>*That dispensary only provides clinic services for children. And even then, I couldn't have told them anything about my PPIUD because it is a Catholic Mission dispensary, they don't deal with FP methods. —Woman 18, postpartum*<br>• **Expectation for remuneration among staff who did not receive initial training**<br>*They will say 'you are the one who received 'Vitamin M' (money) and ate alone, and now you want to just vomit the material to us'.* —Provider<br><br>*There are some who received it well. At this facility, we have a norm that if you go to a training, when you come back you have to provide feedback. So when you're with your fellow staff on shift and you have some time, you can instruct and teach others what you learned and it becomes easy ... but not all staff do this because some may complain, saying 'you got the money and now you come back with just words'.* —Provider

Supplement to: Hackett K, Huber-Krum S, Francis JM, et al. Evaluating the implementation of an intervention to improve postpartum contraception in Tanzania: A qualitative study of provider and client perspectives. *Glob Health Sci Pract*. 2020;8(2). https://doi.org/10.9745/GHSP-D-19-00365

**Outcome**	**Definition**	**Barrier or Facilitator**	**Barrier/Facilitator Identified and Illustrative Quotes**
Sustainability	Long-term maintenance and institutionalization of the PPIUD Initiative	Facilitator	• **Support for population policies and family planning programs to achieve fertility reduction goals**<br>*I don't see any reason why it would fail because we have seen that we have a problem of high population, and we have an intervention that can reduce this population growth rate, so why can't the government support this? ... If they are able to supply other services then why not this as well… it's a national priority and it is in the sustainable development goals!* —Provider
		Barrier	• **Breakdown of supply chain and stock outs**<br>*Once we have many clients ... the need [for PPIUD] will rise but there are few materials, so the project should try to check things that you can support ... we were getting 10 people per month, but now we have started receiving fifty people. Now everything is finished, even the IUDs were few. Once the number of people has increased to fifty it means the need will rise* —Provider<br><br>*To be honest it will be difficult to supply the instruments to other facilities so that they may provide this service… that is not easy and it may take a long time because for the time being, our facility is supplied by donors - that's why it's possible. But with the government, you may write a request for the material until all the ink in that pen is gone, and yet you may not get what you asked for!* —Provider
**Service Outcomes**			

Supplement to: Hackett K, Huber-Krum S, Francis JM, et al. Evaluating the implementation of an intervention to improve postpartum contraception in Tanzania: A qualitative study of provider and client perspectives. *Glob Health Sci Pract*. 2020;8(2). https://doi.org/10.9745/GHSP-D-19-00365

Outcome	Definition	Barrier or Facilitator	Barrier/Facilitator Identified and Illustrative Quotes
Equity	Extent to which the PPIUD implementation is equally available/ accessible to all intended beneficiaries	Barriers	• **Differential treatment by healthcare providers**<br>*Young women and school girls won't waste their time going to clinics for FP methods because the nurses may start questioning why (they) want them. The easiest (place) is those drug shops; you just go in with your money and they give you what you want. There are no questions or records like in the hospitals.* —Woman, postpartum<br><br>*They would prioritize women who came with their husbands. It did not matter whether you were there first, they would ask those who came with their husbands to go in for the services first ... In most cases, my husband is always with me because he knows when you go to the clinic with a man, you will be given priority.* —Woman, postpartum<br>• **Financial barriers to accessing hospitals**<br>*Another challenge is women's economic status. Some give birth here (at the health center) for free, but when you ask her to take a TZS200 bajaji (motorized rickshaw) to [the large hospital], she can't manage it, so we feel that we are going to lose them… and the providers there (at the hospital) are overloaded, so women are scared. They prefer to get the delivery services here… the hospital is very busy.* —Provider<br><br>*I think every woman should be asked where they come from and see how to link them with health providers in the neighboring areas and ask them to be checking on them. For example, I have been asked several times to go to [the hospital] for postpartum, but considering my economic status, I was unable to. It's not because I did not want to go, but because I did not have a starting point even when they told me that my transport costs will be refunded. They should be visiting us in our areas.* —Woman, postpartum<br><br>• **Lack of community-based PPFP counseling and services**<br>*We should also get out of this hospital and educate people in the village because problems are not only in town. A majority who face challenges are in the villages, in our districts… we have to go there and train people. The community should be trained so that they can get awareness… that is my opinion, and if we do this [I believe] people will use these contraceptive methods* —Provider

Supplement to: Hackett K, Huber-Krum S, Francis JM, et al. Evaluating the implementation of an intervention to improve postpartum contraception in Tanzania: A qualitative study of provider and client perspectives. *Glob Health Sci Pract*. 2020;8(2). https://doi.org/10.9745/GHSP-D-19-00365

Outcome	Definition	Barrier or Facilitator	Barrier/Facilitator Identified and Illustrative Quotes

Supplement to: Hackett K, Huber-Krum S, Francis JM, et al. Evaluating the implementation of an intervention to improve postpartum contraception in Tanzania: A qualitative study of provider and client perspectives. *Glob Health Sci Pract*. 2020;8(2). https://doi.org/10.9745/GHSP-D-19-00365

Client receptiveness/ demand for services	Client receptiveness to being counseled on PPFP and/or demand for receiving the PPIUD	Facilitators	• **Level of support from husband/partner**<br>*My husband supported me … When I first told him about the loop, he would always remind me to ask the nurses about it whenever I went to hospital for clinic and he would ask what they said about it. Now when I tell him that I'm going for checkups, he allows me and gives me money for transport. —Woman 2, postpartum*<br>• **Shared intention among couples to space pregnancy for financial reasons**<br>*Depending on my business and the way I planned with my husband, I know that if I use the loop there are some things we will be able to accomplish before getting pregnant again. —Woman 18, prenatal*<br>• **Community and gender norms related to birth spacing**<br>*First, it's because the baby will still be very young, [and] second, I feel shame when I am in the community, getting pregnant when the baby is only about 4 or 5 months old; it's a shameful thing! ... If you happen to get pregnant [again], they tend to put all the weight on women, like 'how did you let yourself get pregnant?' He won't abandon you but he will put all the blame on you, saying that you did it on purpose because you knew that this might happen. —Woman 4, prenatal*<br>• **Community diffusion of preference for PPIUD and peer recommendation**<br>*At some point I was acting like an ambassador for the IUD because many women would come to me; they felt that I understood more. Even the health service providers would tell women to [talk to me] and I would share with them what the IUD was all about. Many women would question how it would be possible for one to have the PPIUD inserted immediately after delivery. They thought it was not possible —Woman 13, postpartum*<br>• **Women's trust in provider advice**<br>*They are the people who know what's going on… people in the street don't know anything about contraceptive issues, I mean scientifically. We know about this by words only, but I believe doctors and nurses are the people who study it. It's their profession, that's why they are the right people to trust when talking about contraception —Woman 13, prenatal*<br>• **Cost-free counseling and insertion services**<br>*I motivate the women and explain the advantages of contraception, and when a woman comes in thinking maybe there are costs to be paid for contraception issues, I explain*

Supplement to: Hackett K, Huber-Krum S, Francis JM, et al. Evaluating the implementation of an intervention to improve postpartum contraception in Tanzania: A qualitative study of provider and client perspectives. *Glob Health Sci Pract*. 2020;8(2). https://doi.org/10.9745/GHSP-D-19-00365

Outcome	Definition	Barrier or Facilitator	Barrier/Facilitator Identified and Illustrative Quotes
			that in our facility the contraceptive services are provided freely, without payment. —Provider 11
		Barriers	Fear of insertion, concerns related to sexual experiences post-insertion, unexpected expulsion and experience of unanticipated side-effects (We have published these results elsewhere).[26]
Satisfaction with PPIUD services	Client receptiveness to being counseled on PPFP and/or receiving PPIUD; Satisfaction with counseling and services	Facilitators	• **Delivery of counseling and services by female provider**<br>*It's the nurses [I trust] because they are also women and when you ask them, many of them have used the method themselves, and they will tell you. But a [male] doctor will only tell you because they studied a lot… they don't have firsthand experience with any method. —Woman 20, postpartum*<br>• **Interpersonal aspects of care**<br>*I am very grateful to them —Providers] because they gave me all the support I needed. They were ready to give answers when I had questions even before I started using the PPIUD, when I had it and when I went to remove it… They have really been supportive, even after removing the PPIUD… calling to ask if I was doing fine and if there is any assistance I needed with family planning methods. —Woman 17, postpartum*

Supplement to: Hackett K, Huber-Krum S, Francis JM, et al. Evaluating the implementation of an intervention to improve postpartum contraception in Tanzania: A qualitative study of provider and client perspectives. *Glob Health Sci Pract*. 2020;8(2). https://doi.org/10.9745/GHSP-D-19-00365

Outcome	Definition	Barrier or Facilitator	Barrier/Facilitator Identified and Illustrative Quotes
		Barriers	• **Perceived provider incompetence**<br>*I never went back [to the health center] because I was scared… The PPIUD getting out of place is not something that I would like to experience again… The providers should be careful when fixing the PPIUD, to avoid incidences like the one I experienced – such incidents could scare women and force them to run from the method, as I did. —Woman 10, postpartum*<br><br>*The person that inserted the PPIUD for me did not insert it well. During the process of inserting, she struggled very much, and it was like a trial and error thing ... It was very painful because I had just given birth. When I got home, the PPIUD was expelled ... The providers giving PPIUD services should be trained [on insertion] and should only be allowed to provide that service when they are competent enough; otherwise, they will make many women run away from using the IUD method. —Woman 1, postpartum*
